# Cancer immunity marker RNA expression levels across gynecologic cancers: Implications for immunotherapy

**DOI:** 10.21203/rs.3.rs-2551645/v1

**Published:** 2023-02-16

**Authors:** Jessica Jou, Shumei Kato, Hirotaka Miyashita, Kartheeswaran Thangathurai, Sarabjot Pabla, Paul DePietro, Mary Nesline, Jeffrey Conroy, Eitan Rubin, Ramez Eskander, Razelle Kurzrock

**Affiliations:** Oregon Health and Sciences; University of California, San Diego Moores Cancer Center; Dartmouth Cancer Center; Ben-Gurion University of the Negev; OmniSeq Inc; OmniSeq Inc; Omniseq; OmniSeq Inc; Ben Gurion University of the Negev; University of California San Diego Moores Cancer Center; University of California, San Diego Moores Cancer Center

## Abstract

**Background:**

Our objective was to characterize cancer immunity marker expression in gynecologic cancers and compare immune landscapes between gynecologic tumor subtypes and with non-gynecologic solid tumors.

**Methods:**

RNA expression levels of 51 cancer-immunity markers were analyzed in patients with gynecologic cancers vs. non-gynecologic cancers, and normalized to a reference population of 735 control cancers, ranked from 0–100, and categorized as low (0–24), moderate (25–74), or high (75–100) percentile rank.

**Results:**

Of the 72 patients studied, 43 (60%) had ovarian, 24 (33%) uterine, and 5 (7%) cervical cancer. No two immune profiles were identical according to expression rank (0–100) or rank level (low, moderate, or high). Patients with cervical cancer had significantly higher expression level ranks of immune activating, pro-inflammatory, tumor infiltrating lymphocyte markers and checkpoints than patients with uterine or ovarian cancer (p<0.001 for all comparisons). However, there were no significant differences in immune marker expression between uterine and ovarian cancers. Tumors with PD-L1 TPS =>1% versus 0% had significantly higher expression levels of pro-inflammatory markers (58 vs. 49%, p=0.0004). Compared to patients with non-gynecologic cancers, more patients with gynecologic cancers express high levels of IDO-1 (44 vs. 13%, p<0.001), LAG3 (35 vs. 21%, p=0.008) and IL10 (31 vs. 15%, p=0.002.)

**Conclusions:**

Patients with gynecologic cancers have complex and heterogeneous immune landscapes that are distinct from patient to patient and from other solid tumors. High levels of IDO1 and LAG3 suggest that clinical trials with IDO1 inhibitors or LAG3 inhibitors, respectively, may be warranted in gynecologic cancers.

## Introduction

Immunotherapies have revolutionized the treatment of solid tumors, with efficacy, even in patients with metastatic disease and multiple lines of prior therapy^1^. Importantly, there is a growing body of literature to support the role of the immune system in the development, response to treatment, and behavior of gynecologic cancers and, hence, immunotherapy has emerged as an area of special focus for these malignancies.

Recent data suggest endometrial cancers can be classified into subtypes that may inform precision genomic and immunotherapies^2^. Endometrial cancers have been found to have one of the highest programmed-death ligand 1 (PD-L1) expression levels^3^ and prevalence of microsatellite instability (MSI) compared to other cancer types^4^. High tumor mutational burden (TMB) and neoantigen load from polymerase epsilon (POLE) mutant and MSI-high (MSI-H) malignancies, each of which can correlate with subgroups of endometrial cancer, associate with increased tumor-infiltrating lymphocytes (TIL)^5^ and improved survival^6^. For these reasons, perhaps, immunotherapy strategies have seen clinical efficacy in endometrial cancers. In 2017, the Food and Drug Association (FDA) issued its first tissue-agnostic approval for pembrolizumab, a programmed cell death protein-1 (PD-1) signal pathway inhibitor, in solid tumors with MSI-H or mismatch repair (MMR) deficiency^7,8,9^, and eventually to all tumors with high TMB (10 mutations/megabase)^10^. The combination of pembrolizumab and a multi-kinase inhibitor lenvatinib was next approved in 2019 for patients with MMR-proficient endometrial tumors^11^. In April 2021, the FDA granted accelerated approval for dostarlimab (anti-PD-1) for the treatment of adult patients with deficient MMR recurrent or advanced endometrial cancer^12^. Ultimately, in March 2022, the FDA also granted approval of pembrolizumab for patients with MSI-H/dMMR advanced endometrial cancer, who have disease progression following prior systemic therapy based on the updated results of KEYNOTE-158^13^.

Persistent human papilloma virus (HPV) infection is believed to cause 99.7% of invasive cervical cancers, making cervical cancer another disease site anticipated to be responsive to immunotherapy, since viral neoantigens may be immunogenic^14^. Moreover, HPV infections have been found to increase PD-L1 expression^15^, thereby creating an immune-privileged environment^16^. In the phase III, KEYNOTE 826 trial, pembrolizumab given upfront with chemotherapy improved both overall survival (OS) and progression-free survival (PFS) in patients with PD-L1 positive cervical cancer^17^, supporting the FDA approval of pembrolizumab in these patients. For PD-L1 negative cervical cancer patients, the combination of ipilimumab (anti-CTLA4) and nivolumab (anti-PD1) demonstrated an objective response rate of 31.6%^18^.

In patients with ovarian cancer, the benefit of immunotherapy is less clear. Given ovarian cancer patients with increased TILs have longer survival^19^, it would seem logical for immunotherapy to have a successful role. However, the response rate to single-agent nivolumab in platinum-resistant patients was only 15%, with no correlation between clinical response and PD-L1 expression level^20^. The incorporation of avelumab, an anti-PD-L1 monoclonal antibody, into frontline chemotherapy in patients with ovarian cancer also failed to show efficacy^21^. While a higher objective response rate (ORR) has been observed in ovarian tumors with PD-L1 immunohistochemistry (IHC) combined positive score (CPS) 10% compared to patients with CPS <10% (17.5 v 8%)^22^, the ORRs for both are low. Furthermore, in the placebo controlled, randomized, phase 3 IMagyn050/GOG 3015/ENGOT-OV39 trial, the addition of atezolizumab to platinum-based combination chemotherapy and bevacizumab failed to show an improvement in oncologic outcomes^23^. Overall, there is an impression that the immunosuppressive tumor microenvironment in patients with ovarian cancer is difficult to overcome with a single-agent approach.

There is a strong rationale for using immunotherapy in patients with gynecologic tumors, but the efficacy of this approach is limited in part by our understanding of the biologic/immune underpinning of these cancers. In our study, we utilized RNA-seq immune gene expression to begin describing the immune pressures in ovarian, uterine, and cervical tumors. Our findings may begin to explain the differential responses to immunotherapies between gynecologic disease sites and compared to non-gynecologic malignancies.

## Materials And Methods

### Patients

Cancer-immunity markers among 72 eligible, consecutive patients with gynecologic solid cancers seen at the University of California San Diego Moores Cancer Center for Personalized Therapy were evaluated at a Clinical Laboratory Improvement Amendments (CLIA)-licensed and College of American Pathologist (CAP)-accredited clinical laboratory, OmniSeq (https://www.omniseq.com/). All investigations followed the Institutional Review Board protocol for data collection (Profile Related Evidence Determining Individualized Cancer Therapy, NCT02478931) and for any investigational interventions for which the patients consented.

### Tissue samples and analysis of cancer-immunity markers

Formalin-fixed, paraffin-embedded (FFPE) tumor specimens were evaluated with RNA sequencing by OmniSeq laboratory. Total RNA was extracted utilizing the truXTRAC FFPE extraction kit (Covaris, Inc., Woburn, MA), following the manufacturer’s instructions with modifications as needed. After purification, RNA was eluted in 50 μL water and yield was assessed by the Quant-iT RNA HS Assay (Thermo Fisher Scientific, Waltham, MA), as per manufacturer’s recommendation. A predefined yield of 10 ng RNA was considered acceptable to ensure library preparation. RNA-sequencing absolute reads were generated using the Torrent Suite’s plugin immuneResponseRNA (v5.2.0.0). The RNA expression of 51 targeted immune response genes were assessed as follows: 9 checkpoints (PD-1, PD-L1, PD-L2, BTLA, CTLA-4, LAG3, TNFRSF14, TIM-3, and VISTA); 3 metabolic immune escape markers (ADORA2A, IDO1, and CD39); 2 anti-inflammatory response markers (IL10 and TGFB1); 5 macrophage-associated markers (CCL2, CCR2, CSF1R, CD163, and CD68); 15 T-cell priming markers (GITR, CD137, ICOS, OX40, CD27, CD28, CD80, CD86, CD40, CD40 ligand, ICOS ligand, OX40 ligand, GZMB, IFNG, and TBX21); 7 pro-inflammatory response markers (IL1B, MX-1, STAT1, TNF, DDX58, CXCL10, CXCR6); 8 tumor infiltrating lymphocyte markers (CD4, CD8, FOXP3, CD2, CD3, KLRD1, SLAMF4, and CD20); and other immunotherapy markers (CD38 and GATA3) (Supplemental Table 1 for list and function information^24^). Transcript abundance was normalized and compared to an internal reference population (735 patients with a range of solid tumors). Rank values were set on a scale of 0 to 100 and percentile rank levels were categorized as “High” (75 – 100), “Moderate” (25–74), “Low” (0–24). Checkpoint markers, macrophage associated markers, anti-inflammatory markers and metabolic immune escape markers were considered “immune suppressive markers.” T-cell primed markers and pro-inflammatory response markers were considered “immune activating markers.”

**Figure 1:**
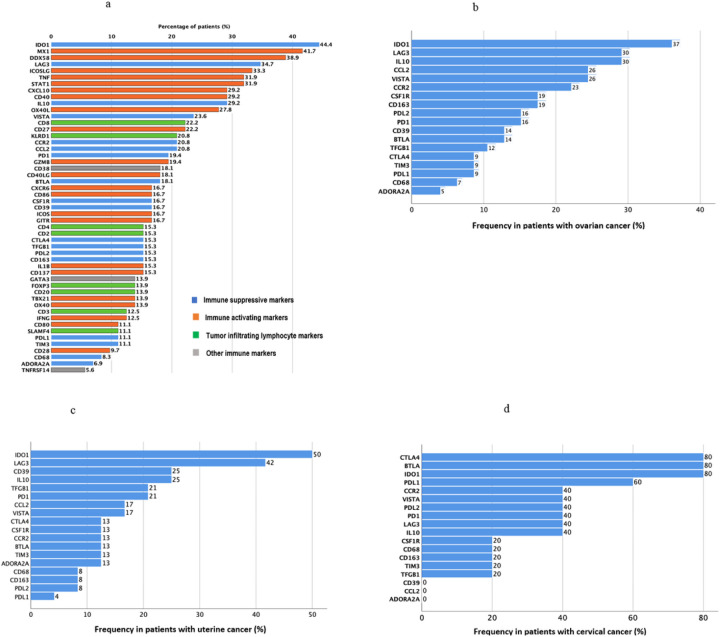
**a.** Frequency of high RNA expression (≥75 percentile rank) among cancer-immunity markers across gynecologic disease sites (see also Supplemental Table 1 and Methods) (N=72) **b.** Frequency of high RNA expression (≥75 percentile rank) among immune suppressive markers in patients with ovarian cancer (see also Supplemental Table 1 and Methods) (N=43) **c.** Frequency of high RNA expression (≥75 percentile rank) among immune suppressive markers in patients with uterine cancer (see also Supplemental Table 1 and Methods) (N=24) **d.** Frequency of high RNA expression (≥75 percentile rank) among immune suppressive markers in patients with cervical cancer (see also Supplemental Figure 1 and Methods) (N=5)

**Figure 2:**
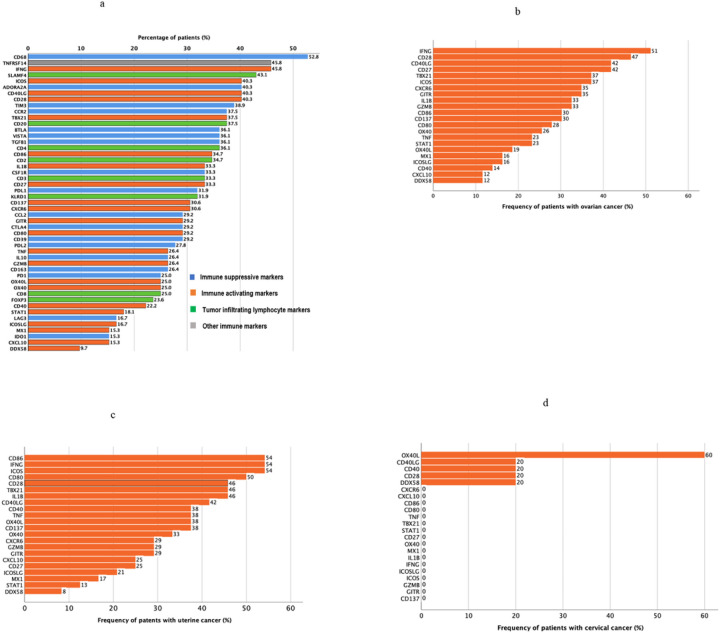
**a.** Frequency of low RNA expression (<25 percentile rank) among cancer-immunity markers across gynecologic disease sites (see also Supplemental Table 1 and Methods) (N=72 **b.** Frequency of low RNA expression (< 25 percentile rank) among immune activating markers in patients with ovarian cancer (see also Supplemental Table 1 and Methods) (N=43) **c.** Frequency of low RNA expression (< 25 percentile rank) among immune activating markers in patients with uterine cancer (see also Supplemental Table 1 and Methods) (N=24) **d.** Frequency of low RNA expression (< 25 percentile rank) among immune activating markers in patients with cervical cancer (see also Supplemental Figure 1 and Methods) (N=5)

Frequency of patients with high RNA expression level ranks (RNA expression levels ≥75^th^ percentile rank) and low RNA expression level ranks (RNA expression levels <25^th^ percentile rank) were calculated and shown on bar graphs (Figures 1a and 2a).

PD-L1 protein expression was measured using the tumor proportional score (TPS) (percentage of viable tumor cells showing partial or complete membrane IHC staining of any intensity). The expression of PD-L1 on the surface of tumor cells was assessed via the Dako Omnis platform (Agilent, Santa Clara, CA) using the anti-PD-L1 22C3 pharmDx antibody (Agilent, Santa Clara, CA), and expression levels were scored as per manufacturer guidelines.

Immunoprints refer to an overview of each individual patient’s immune Profile. Each column represents a unique patient and each row represents the immune markers investigated. The corresponding cell is colored according to RNA expression level rank: high, moderate, or low.

An immunogram is an overview of immune profiles across patient cohorts. Immune markers were categorized according to their function, to one of six categories: immune checkpoints, immune escape/anti-inflammatory markers or macrophage associated markers, tumor infiltrating lymphocyte markers, pro-inflammatory markers, and T-cell primed markers^25^. Each immune marker category makes up each “spoke” of the radar plot. The length of each spoke corresponds to the average RNA transcript rank expression level (0–100). The mean RNA expression level rank of immune factors in each immune marker category was plotted on each spoke and connected to visually compare the “immune pressures” between disease sites and cohorts^26–29^.

### Endpoints and statistical methods

Patient characteristics and the pattern of cancer-immunity markers were summarized by descriptive statistics. Proportions were compared using Fisher’s Exact test. Means were compared using Student’s t test. The correlation between the disease sites and comprehensive expression patterns was evaluated through principal component analysis (PCA) and quantified by calculating silhouette scores. Statistical analyses were performed using SPSS version 28.0 (Chicago, IL, USA), Microsoft Excel version 16.49, and R 3.6.1 (R Foundation for Statistics Computing, Vienna, Austria). A p value ≤0.05 was considered significant.

## Results

### Patient and tumor characteristics

Clinicopathologic features of our cohort of 72 patients with gynecologic cancers are depicted in Table 1. Forty-three patients (60%) had ovarian cancer; 24 (33%) uterine cancer; and 5 (7%) cervix cancer. Of the cohort of patients with ovarian cancer, 52% were less than 65 years of age, no tumors had TMB ≥10 mutations/megabase, and 49% had tumors with a PD-L1 TPS of ≥1%. Of the cohort of patients with uterine cancer, 46% were less than 65 years of age, 8% (N= 2) had tumors with TMB ≥10 mutations/megabase (both of which were MSI-High), and 37% had tumors with a with a PD-L1 TPS of ≥1%. Of the small cohort of patients with cervix cancer (N=5), the majority (N=4) were less than 65 years of age, no patient had a tumor with TMB ≥10 mutations/megabase and 2 patients had a PD-L1 TPS score that was positive.

**Figure 3:**
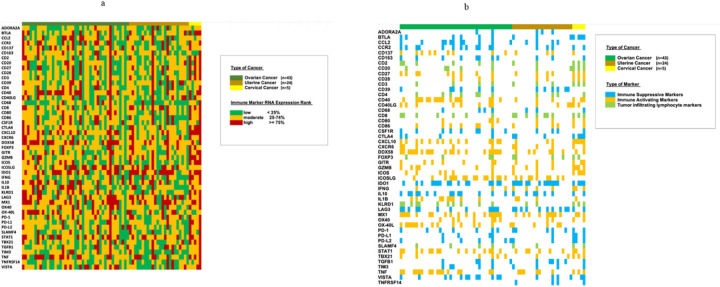
**a.** Immunoprint showing on overview of RNA expression level of multiple immune markers for each individual case (N=72). Percentile rank of transcriptomic expression was determined by normalizing to RNA of 735 control patients with diverse solid tumors. Each column indicates individual patients’ case. (See also Supplemental Table 1 and Methods). This figure shows that no two patients had the same immune marker expression pattern. **b.** Immunoprint showing an overview of high (>=75 percentile rank) RNA expression level of multiple immune markers for each individual case (N=72). Each column indicatesan individual patients’ case. See also Supplemental Table 1 and Methods for classification of immune markers as immune suppressive, immune activating, or tumor infiltrating lymphocyte markers. This figure shows that no two patients had the same pattern of high RNA expression.

**Figure 4:**
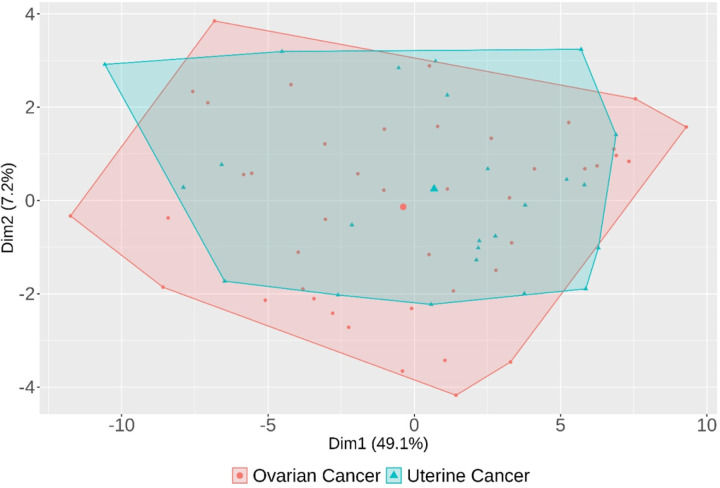
Cluster plot of principal component analysis for 51 cancer-immunity marker RNA expression ranks in patients with ovarian and uterine cancer (n=72) (see also Supplemental Figure 1 and Methods). Largely overlapping clusters suggest that expression patterns of cancer-immunity markers were not associated with disease site. The principal component analysis summarizes 51-dimensional data corresponding to different cancer-immunity markers on a two-dimensional field to analyze the distribution of the samples. The first dimension is the vector that represents the greatest variation in the data (“Dim1”), which captures 49.1% of the variation in the data. The second dimension (“Dim2”) represents 7.2% of the variation in the data. Together, both vectors capture 56.3% of the variation. The cluster assignment is based on the site of disease (pink distribution represents ovarian cancer vs. green distribution represents uterine cancer.) Each symbol in the field represents each case of uterine or ovarian cancer. Patients with cervical cancer were excluded from the analysis due to small numbers. A clear separation in distribution (pink vs. green colors) would suggest that immune marker expression patterns were dependent on disease site. Overlapping distribution would suggest that expression patterns were independent of disease site. From this plot, the distribution cancer immunity marker expression for patients with uterine and ovarian cancer were largely overlapping, which suggests that the pattern of RNA expression pattern of 51 markers were not associated with disease site.

**Figure 5:**
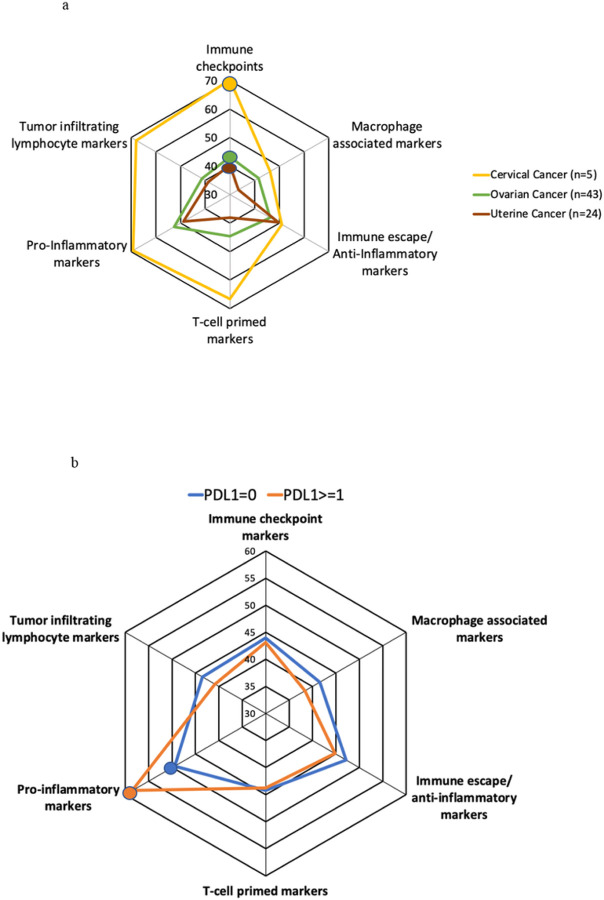
**a.** Sample immunogram of mean cancer-immunity marker RNA expression rank in patients with gynecologic cancers (N=72) relative control cancer types (n=735) (See also Supplemental Table 1). As an example, the average RNA expression rank level of immune checkpoint markers in ovarian cancers (green line) is higher than 43% (green circle) of other cancer types. For uterine cancers (brown line), average RNA expression rank level of immune checkpoint markers is higher than 40% (brown circle) of other cancer types. For cervical cancer (yellow line), average RNA expression rank level of immune checkpoint markers is higher than 70% (yellow circle) of other cancer types; immune activating markers (tumor infiltrating lymphocyte markers and pro-inflammatory markers) are also high in cervix cancers compared to other cancer types. Although a small sample size, this figure demonstrates that cervical cancers have significantly higher expression rank levels of immune checkpoints, tumor infiltrating markers, pro-inflammatory markers and T-cell primed markers compared to ovarian and uterine cancers (p<0.0001 for all comparisons). Gynecologic cancers express similar levels of immune escape markers and macrophage associated markers. There were no significant differences in mean expression levels of immune marker categories between uterine and ovarian cancers. **b.** Sample immunogram of mean cancer-immunity marker RNA expression rank in gynecologic cancers by PDL1 status IHC (tumor proportion score) (see also Supplemental Table 1) (N=72). In this immunogram, gynecologic tumors with PDL1 staining >=1% express significantly higher levels of pro-inflammatory markers (orange circle) compared to tumors with PDL1 scores of 0% (blue circle) (58 vs. 49%, p=0.0004). There were no other statistically significant differences in other immune categories by PDL1 staining. PDL1 staining may be associated with pro-inflammatory markers of the cancer immunity cycle.

The immune profiles of the patients varied between patients and disease and are shown in Figures 1–5, Supplemental Figure 1, Tables 1 and 2.

### Multiple potentially actionable immune inhibitory and immune stimulatory markers were expressed in gynecologic malignancies

Both immune suppressive markers and immune activating markers were examined. High levels of immune suppressive markers are relevant as they can be counteracted by agents that inhibit them. Low levels of immune activating markers are relevant as they would need to be augmented in a therapeutic setting. Figure 1a summarizes the percentage of patients with gynecologic cancers that had high RNA expression of each immune marker. Among immune suppressive markers, patients with gynecologic cancers were most likely to have high expression of IDO1 (44%), LAG3 (35%), IL10 (29%), VISTA (24%), CCR2 (21%), CCL2 (21%), PD1 (19%). Among immune activating markers, patients with gynecologic cancers were most likely to have high expression of MX1 (42%), DDX58 (39%), ICOSL (33%), TNF (32%), STAT1 (32%), CXCL10 (29%), CD40 (29%), and OX40L (28%). Among tumor infiltrating lymphocyte markers, patients with gynecologic cancers were most likely to have high expression of CD8 (22%) and KLRD1 (21%). Figure 2a depicts the percentage of patients with gynecologic cancers that had low RNA expression of each immune marker. For example, among immune activating markers, patients with gynecologic cancers were most likely to have low expression of IFNG (43%), ICOS (40%), CD40LG (40%), CD28 (40%), and TBX21 (38%). Among immune suppressive markers, patients with gynecologic cancers were most likely to have low expression of CD68 (53%), ADODRA2A (40%), TIM3 (39%), and CCR2 (38%).

The percentage of patients with high RNA expression of immune suppressive markers within gynecologic disease sites is further depicted in Figure 1b–d. The most common highly expressed immune suppressive markers in patients with ovarian cancer are IDO1 in 37% of patients, LAG3 (30%), and IL10 (30%) (Figure 1b). In patients with uterine cancer, high expression levels of IDO1 is again seen in 50% of patients and high expression levels of LAG3 seen in 42% of patients (Figure 1c). Four of five patients with cervix cancer express high levels of CTLA4, BTLA and IDO1 and three of five patients express high levels of PDL1 (Figure 1d).

The percentage of patients with low RNA expression of immune activating markers within gynecologic disease sites is further depicted in Figure 2a–d. In patients with ovarian cancer, low levels of IFNG are seen in 51% of patients, CD28 (47%), CD40LG (42%), and CD27 (42%) (Figure 2b). In patients with uterine cancer, 54% of patients had low expression levels of CD86, IFNG and ICOS (Figure 2c). In patients with cervical cancer, three of five patients had low RNA expression levels of OX40L, and one of five had low expression of CD40LG, CD40 CD28 and DDX58 (Figure 2d).

### IDO1, an immune suppressive marker, is the transcript most frequently highly expressed in gynecologic cancers

Figure 1a demonstrates that 44.4% of gynecologic cancers had high expression of the immunosuppressive IDO1 RNA. IDO1 was highly expressed in 37% of ovarian cancers, 50% of uterine cancers, and 4 of 5 patients with cervical cancer (Figures 1b, c, d).

### LAG3, an immunosuppressive marker, is highly expressed in ovarian and uterine cancers

LAG3 was the second most frequently expressed immunosuppressive marker in ovarian (30% of cases) and in uterine cancer (42% of cases). In cervical cancer, it was highly expressed in 2 of 5 patients (Figures 1a–3d).

### A significantly higher proportion of patients with gynecologic cancers express high levels of IDO-1, LAG3 and IL10 compared to non-gynecologic solid tumors.

For immune inhibitory factors, the proportion of patients with high expression in gynecologic malignancies (N=72) was compared to those with high expression in non-gynecologic malignancies (N=442 patients Profiled at UCSD). Potential drugs would be those agents that suppress high expression of immune inhibition.

Thirty-two of 72 (44%) patients with gynecologic cancers expression high levels of IDO-1 compared to 13% of patients with non-gynecologic cancers (p<0.0001) (Table 2). When IDO-1 is high, it can potentially be targeted with IDO1 inhibitors such as epacadostat, indoximod, etc.

Patients with gynecologic cancers also have high expression levels of LAG3 (35% vs. 21%, p=0.008), compared to patients with non-gynecologic solid tumors. When LAG3 is high, it is potentially targetable with LAG3 inhibitors such as relatlimab, BI 754111, LAG525 and MK-4280.

Patients with gynecologic cancers also have high expression levels of IL10 compared to those with non-gynecologic cancers (31% vs. 15%, p=0.002). When IL10 levels are high, they are potentially targetable with IL10 inhibitors such as MK-1966 (see Supplemental Table 1).

A significantly smaller proportion of patients with gynecologic cancers express high levels of ADORA2A as compared to other cancers (6.9% versus 23.1%; p=0.002), which may mean less success with using drugs that target this protein in this patient population.

For immune-stimulatory factors, we similarly compared the proportion of patients with low expression in those with gynecologic malignancies to those with non-gynecologic malignancies. Potential drugs would be agents that stimulate the specific immune stimulatory function. There were no statistically significant differences in proportion of patients with low expression of immune stimulatory factors (ie. IFNG, ICOS, CD40LG, IL1B, CD137, CITR, TNF, OX40L, OX40, ICOSLG) between those with gynecologic cancers and those with non-gynecologic cancers.

### Immune marker RNA expression level differed from patient to patient amongst 72 individuals with gynecologic cancer

Fifty-one immune markers were investigated. Figure 3a depicts an immunoprint of RNA expression levels (low=<25%, moderate 25–74%, or high >=75%) across immune markers for each individual patient studied. Figure 3b depicts an immunoprint of only high RNA expression levels (>=75%) across immune markers. There were no two gynecologic cancers with identical immune portfolios according to immune RNA expression rank numbers (1–100) or according to immune expression rank levels (low, moderate, or high).

### Immune marker RNA expression level could not be clustered based on uterine of ovarian histology.

A cluster plot withprincipal component analysis (Figure 4) that summarized 51-dimensional data corresponding to the 51 different cancer-immunity markers on a two-dimensional field demonstrated that the distribution cancer immunity marker RNA expression for patients with uterine and ovarian cancer were largely overlapping, which suggests that the pattern of RNA expression pattern of 51 markers were not associated with disease site. (Since there were only 5 patients with cervical cancer, these tumors were not mapped on this plot). The finding was validated by the calculation of silhouette score, which represents the variation within clusters compared to the variation between clusters. The silhouette score was 0.011, while the silhouette score of one million times of randomization of cancer sites in the same cohort was calculated as 0.00 ± 0.012. (mean and error). This suggests that the comprehensive expression patterns of 51 genes did not correlate with disease site.

### Patients with cervix cancer, but not those with ovarian or uterine cancer, have higher RNA expression level ranks of immune checkpoint, TIL, pro-inflammatory and T-cell primed markers than approximately 70% of patients with other cancer types.

An immunogram was generated by plotting the average RNA expression level rank of immune markers for each corresponding immune marker category on a radar plot (Figure 5a). Although the sample size is small, cervical cancers have a mean RNA expression rank level of immune checkpoint, TIL, pro-inflammatory and T-cell primed markers higher than ovarian and uterine cancers (p<0.0001 for all comparisons) and higher than approximately 70% of all other cancer types. Cervix cancers have an average RNA expression rank level of immune escape/anti-inflammatory markers (51% rank), and slightly less than average (47%) rank level of macrophage associated markers compared to other cancer types.

Ovarian and uterine cancers have lower than average (<50% rank level) expression of almost all immune categories including immune checkpoints, TIL, T-cell primed and macrophage associated markers compared to other cancer types. Ovarian cancers have a slightly higher than average RNA expression level (52%) of pro-inflammatory markers compared to other cancer types. There were no significant differences in mean expression levels of any immune marker categories between uterine and ovarian cancers.

### Patients with PD-L1 TPS >= 1% have significantly higher mean RNA expression rank levels of pro-inflammatory markers than those with PD-L1 TPS of 0%.

An immunogram was next generated to depict mean RNA expression levels of each immune category by PD-L1 IHC status (Figure 5b). Patients with PD-L1 IHC TPS >=1% had significantly higher expression levels of pro-inflammatory markers compared to those with PD-L1 IHC TPS 0% tumors (58 vs. 49%, p=0.0004). Immunograms of each disease site by PDL1 status are shown in Supplemental Figure 1a-c.

## Discussion

In our report, we characterize the immune profiles of patients with gynecologic cancers using 51 RNA transcript levels associated with the cancer immunity cycle. Notably, we found that no two tumors have an identical immune Profile. This finding highlights the complexities of immune interactions, as well as the need to interrogate each tumor in the context of choosing precision immunotherapeutics. Contemporary work with The Cancer Genome Atlas (TCGA) and other large databases, integrating immunogenomics using powerful computational science have started to describe six distinct clusters of immune subtypes with potential implications for cancer treatment, though the prior work also demonstrated significant interpatient immune landscape heterogeneity^30,31^. These unique immune profiles again highlight the opposing immune pressures that affect the tumor microenvironment. Moreover, patient and tumor heterogeneity have implications for treatment choice. It remains challenging to design clinical trials in the context of big data and next generation sequencing, the latter which also suggests that the molecular genomic Profile of metastatic tumors differ from patient to patient; however, emerging observations, at least in the precision genomics space, suggest that treatment regimens based on analyzing each patient’s cancer genomic landscape using next generation sequencing may improve patient outcomes^32,33,34^. A similar paradigm of individual tumor immune Profile interrogation and matching cancers with the right immunotherapy may be required.

We used an immunogram as the framework to describe the interacting immune pressures mentioned previously^26^. Of interest, patients with cervix cancer (though the numbers of patients were small) have higher RNA expression levels of immune-activating factors than immune-inhibitory factors, which may signify a generally “hotter” tumor. Moreover, patients with cervix cancer have higher RNA expression levels of immune-activating factors compared to many other types of solid tumors including uterine and ovarian cancers, consistent with larger studies utilizing the TCGA databases of diverse solid tumors^29^. Patients with uterine and ovarian cancers have a slightly lower than average (<50% expression rank level) RNA level of both immune activating and immune inhibitory markers. These findings may begin to explain the success of incorporating pembrolizumab (anti-PD-1) in the treatment of metastatic cervical cancer, and the comparatively lackluster response with the incorporation of immune checkpoints for patients with ovarian cancer^17,35^.

Upregulation of alternative checkpoints may also explain why some patients do not respond to anti-PD-L1/anti-PD-1 agents^24^. An analysis of immune gene expression in patients with cervix cancer using the TCGA and GEO databases was able to delineate high-versus low-risk immune profiles that reliably predicted survival. The high-risk group was characterized by over-expression of macrophages and mast cells, probably due to their ability to promote lymphangiogenesis and angiogenesis^36^. In our study, we found higher expression levels of pro-inflammatory markers in patients with PD-L1 IHC positive tumors across all three disease sites, but lower levels of other immune markers. This may begin to explain why PD-L1 IHC serves as a limited therapeutic biomarker as it likely captures only one aspect of the immune microenvironment. The IHC 22C3 antibody only measures tumor PD-L1 whereas IHC SP142 measures tumoral and immune cell PD-L1. In patients with cervical cancer, those with PDL1-positive tumors had higher levels of TIL markers as well. However, in patients with ovarian and uterine cancers, PD-L1 positivity did not correlate with higher TIL markers. Perhaps this again explains, at least in part, the unique success of using checkpoint inhibitors in patients with cervical cancer, but not in ovarian cancers.

On the other hand, uterine cancers are also sensitive to anti-PD1 checkpoint blockade, and this might be due to other factors, such as the presence of MSI-High, high TMB and POLE mutations^2,37,38^.

We also investigated over-expression of immune inhibitory factors and under-expression of immune activating factors in gynecologic vs. non-gynecologic tumors with the hypothesis that drugs that block inhibition and or stimulate activation factors may be good candidates for therapeutics. Of interest, patients with gynecologic cancers had higher IDO1 RNA expression levels compared to other cancer types, with about 44% of gynecologic cancers expressing high IDO1 compared to less than 13% of other cancer types (p<0.001). IDO1 is the first and rate-limiting enzyme in the degradation of tryptophan, which is expressed in cancer cells or draining lymph nodes^39^. When IDO1 is high, it can potentially be targeted with IDO1 inhibitors such as epacadostat, indoximod, etc. Previously, bioinformatics analysis of IDO1 immune function in gynecologic cancers using databases such as Oncomine, GEPIA etc. also found over-expression of IDO1 RNA as well as protein expression in gynecologic tumors^40^. Though phase 1 studies showed promising tolerance and response rates to the IDO inhibitor epacadostat^41^, phase 2 studies comparing the IDO inhibitor epacadostat against tamoxifen in ovarian cancer types did not show significantly improved efficacy^42^. IDO1 inhibitors have also failed in other tumor types^43^. However, none of these clinical trials were designed to select patients with specific biomarkers (such high IDO RNA expression levels) for response. Since only a minority of patients (13% in our series) with non-gynecologic cancers have high IDO transcript levels, these results suggest that selection of patients with higher IDO1 levels for IDO1 inhibitor trials may be warranted. Moreover, since almost half of gynecologic cancers express high IDO1 levels, gynecologic malignancies may be a worthwhile target for IDO1 inhibitor studies. The clinical utility of the IDO inhibitor epacadostat in combination with pembrolizumab (NRG GY016) showed a promising ORR in a small cohort of pretreated clear cell ovarian cancer patients (ORR 21%), although the study was terminated prematurely due to lack of drug (Gien et al. IGCS 2022)

We also found higher expression of the checkpoint LAG3 in gynecologic cancers as compared to non-gynecologic cancers (34.7% versus 20.6%; p=0.008); similar findings were previously shown in endometrial cancers^44^. The anti-LAG3 relatlimab was recently FDA approved for melanoma^45^ and other LAG3 inhibitors are in clinical trials. It is plausible that LAG3 may be an alternative checkpoint upregulated in some of the tumors resistant to anti-PD1/PDL1 agents, and LAG3 inhibitors merit investigation in patients with gynecologic cancers, especially those with high LAG3 expression.

There were several limitations to our study. First, our sample size was relatively small, especially in regard to cervical cancer, with uneven distribution among gynecologic disease sites. Second, since the database was not clinically annotated, we were not able to associate immune biomarker expression levels with immunotherapeutic responses or other clinical oncologic endpoints. Though this data was obtained from a CLIA-licensed laboratory, immune marker RNA from normal tissues were not delineated. Finally, while we were able to delineate differences in immune landscape between PDL1-positive versus -negative cancers, the small number of neoplasms with high TMB or MSI-High disease precluded analysis of these subsets. Despite these limitations, this study provides comprehensive insight into the complexities of the immune landscape in gynecologic malignancies

Proteomic and genomic based biomarkers such as MMR, MSI, PDL-1 and TMB have demonstrated efficacy in predicting treatment response to immunotherapies^37,46,47^. RNA sequencing may be an opportunity for discovering a broader cadre of biomarkers to improve the precision and limit the toxicities of this drug class^48,49^. RNAseq provides valuable information such as transcript abundance, molecular alterations and alternative promoter/splice sites that may be used to predict response or resistance to immune therapies^50, 48^. In fact, one study found that almost 90% of patients with (classic) biomarker-negative tumors (MMR proficient, MSI stable, PDL1 <1%, TMB <10mut/mb), had high levels of other immune marker RNA that were potentially targetable with drugs under active investigation^51^. Overall, our study indicates that patients with gynecologic cancers have complex immune landscapes that differ from patient to patient even within the same histology, but that certain pharmacologically tractable immune markers, such as high levels of IDO1 and LAG3 are present in these cancers.

## Figures and Tables

**Table 1.** Clinicopathologic variables among gynecologic disease sites (N=72)

	Ovarian (n=43, (%))	Uterine (n=24, (%))	Cervix (n=5, (%))
Age (years)			
<65	22 (51.2)	11 (45.8)	4 (80.0)
65	21 (48.8)	13 (54.2)	1 (20.0)
TMB (mutations/megabase)			
10	41 (95.3)	19 (79.2)	4 (80.0)
10	0 (0)	2 (8.3)	0 (0)
Unknown	2 (4.7)	3 (12.5)	1 (20.0)
MSI status†			
Low/Stable	35 (81.4)	19 (79.2)	3 (60.0)
High	0 (0)	2 (8.3)	0 (0)
Unknown	8 (18.6)	3 (12.5)	2 (40.0)
PDL-1 IHC (%) ‡			
0	22 (51.2)	15 (62.5)	3 (60.0)
1	8 (18.6)	5 (20.8)	1 (20.0)
2–9	9 (20.9)	2 (8.3)	0 (0)
10–50	4 (9.3)	1 (4.2)	1 (20.0)
>50	0 (0)	1 (4.2)	0 (0)

† MSI-H defined as instability in at least 2 of 5 tested microsatellites

‡ PD-L1 testing by IHC antibody 22C3 testing (percentage by tissue proportion score)

Abbreviations: IHC, immunohistochemistry; MSI, microsatellite instability; PDL-1, programmed death ligand-1; TMB, tumor mutational burden

**Table 2.** Proportion of high (>=75 percentile rank) RNA expression of immune inhibitory immune markers among gynecologic cancers (n=72) compared to non-gynecologic solid tumors (n=442)*. Potential drugs would be those agents that suppress the specific immune inhibitory function.

	Gynecologic cancers (n=72, (%))	Non-gynecologic cancers (n=442,(%))* (%))*	p	Potential Drugs
Immune inhibitory factors^1^				
IDO1	32 (44.4)	58 (13.1)	**<0.001**	When IDO1 is high, it is potentially targetable with IDO1 inhibitors. Examples include: Epacadostat Indoximod BMS-986205 KHK2455 LY3381916
LAG3	25 (34.7)	91 (20.6)	**0.008**	When LAG3 is high, it is potentially targetable with LAG3 inhibitors. Examples of LAG3 inhibitors include: Relatlimab BI 754111 LAG525 MK-4280
IL10	22 (30.6)	68 (15.4)	**0.002**	When IL-10 is considered as an immunosuppressive cytokine, it is potentially targetable with IL-10 inhibitors such as: MK-1966
CSF1R	12 (16.7)	103 (23.3)	0.213	
VISTA	17 (23.6)	148 (33.5)	0.096	
CCR2	15 (20.8)	102 (23.1)	0.186	
PD1	14 (19.4)	79 (17.9)	0.094	
CTLA4	11 (15.3)	76 (17.2)	0.691	
PDL2	11 (15.3)	89 (20.1)	0.910	
PDL1	8 (11.1)	59 (13.3)	0.265	
TIM3	8 (11.1)	81 (18.3)	0.134	
ADORA2A	5 (6.9)	102 (23.1)	**0.002**	

* N=442 non-gynecologic cancers Profiled at UCSD

## Data Availability

Data are available on reasonable request. All data relevant to the study are included in the article or uploaded as online supplemental information.

## What Is Already Known On This Topic:

Gynecologic cancers have differential responses to immunotherapies, dependent on disease site and biomarkers. Immune checkpoint inhibitors are FDA approved in the frontline setting for cervical cancer patients who have PDL1 positive tumors, and for patients with recurrent endometrial cancer with tumors that are mismatch repair deficient or have high tumor mutational burdens. Despite these approvals, these biomarkers do not account for all tumor responses, and often lead to resistance. Furthermore, immunotherapies have not been found to be effective in patients with ovarian cancer. This raises the question of whether other immune mechanisms are at work within the tumor microenvironment that can enhance or hinder its response to immunotherapy.

## What this study adds:

In this study, we found that patients with cervical cancer have significantly higher RNA expression levels of immune-activating, pro-inflammatory and tumor infiltrating lymphocyte markers compared to patients with uterine or ovarian cancers. PDL1 positive tumors also have higher expression levels of pro-inflammatory markers compared to PDL1 negative tumors. Furthermore, gynecologic tumors have a significantly higher expression of IDO-1 markers, which are potentially actionable with IDO inhibitors. Though these findings may begin to explain the success of immunotherapies with these particular characteristics, we do not yet know how to interpret RNA expression levels of both inflammatory and inhibitory markers together. We therefore propose the concept of an “immunogram”, to describe and graph the immune pressures within the tumor.

## How this research might affect research, practice or policy:

By interpreting both the immune stimulatory and inhibitory markers together, we may begin to have a more comprehensive understanding of the tumor microenvironment. Our proposed “immunogram” may provide a framework of how to visualize these immune pressures within each individual tumor. A personalized immunogram may potentially be used to inform treatment decisions in the age of precision oncology.
